# Dexamethasone enhances CD163 expression in porcine IPKM immortalized macrophages

**DOI:** 10.1007/s11626-020-00535-5

**Published:** 2021-01-14

**Authors:** Takato Takenouchi, Takeya Morozumi, Emi Wada, Shunichi Suzuki, Yasutaka Nishiyama, Shin Sukegawa, Hirohide Uenishi

**Affiliations:** 1 Division of Animal Sciences, Institute of Agrobiological Sciences, National Agriculture and Food Research Organization, 1-2 Ohwashi, Tsukuba, Ibaraki 305-8634 Japan; 2 Research & Development Center, NH Foods Ltd., 3-3 Midorigahara, Tsukuba, Ibaraki 300-2646 Japan

## Abstract

In our previous study, we established a unique porcine macrophage cell line, immortalized porcine kidney-derived macrophages (IPKM). The purpose of the present study was to further elucidate the characteristics of IPKM. CD163 is a scavenger receptor for the hemoglobin-haptoglobin complex and is used as a phenotypic marker of anti-inflammatory M2 macrophages. The expression of CD163 is enhanced by dexamethasone (DEX), a potent steroidal anti-inflammatory drug, in human and rodent macrophages in vitro. Therefore, we investigated the effects of DEX on CD163 expression in porcine IPKM. Treatment with DEX markedly enhanced CD163 expression in the IPKM. In addition, we found that SB203580, a selective inhibitor of p38 mitogen-activated protein kinase (MAPK), blocked the effects of DEX, suggesting that the p38 MAPK signaling pathway is involved in the regulation of the DEX-induced enhancement of CD163 expression. Since CD163 is considered to be a putative receptor for the porcine reproductive and respiratory syndrome virus (PRRSV), the effects of DEX on the infection of IPKM by PRRSV were evaluated. Although the IPKM were susceptible to infection by the Fostera PRRSV vaccine strain, DEX treatment did not affect the propagation of the virus in the IPKM. This suggests that the DEX-induced enhancement of CD163 expression alone is not sufficient to facilitate the infection of IPKM by PRRSV.

## Introduction

Macrophages are classical innate immune cells, which play a protective role in host defense against invading pathogens. They work through unique mechanisms, such as phagocytosis, activating immune cells by inducing cytokine secretion, and antigen presentation. Macrophages can be broadly divided into two phenotypes, i.e., classically activated pro-inflammatory M1 and alternatively activated anti-inflammatory M2 subtypes (Murray 2017). They are also recognized as a common target for various viral and bacterial pathogens, and contribute to the infection processes of those pathogens (Finlay and McFadden 2006). Therefore, in vitro systems involving cultured macrophages are useful models for studying pathogen infections and innate immune responses.

Recently, we successfully established a novel porcine macrophage cell line (immortalized porcine kidney-derived macrophages: IPKM) by transferring the SV40 large T antigen and porcine telomerase reverse transcriptase genes using lentiviral vectors (Takenouchi *et al.* 2017). IPKM retain multiple macrophage functions, and thus, might be useful for investigating the interactions between porcine macrophages and various porcine viruses, including the porcine reproductive and respiratory syndrome (PRRS) and porcine epidemic diarrhea viruses.

Several cell surface receptors have been found to be involved in the infection of macrophages by the PRRS virus (PRRSV) (Zhang and Yoo 2015). Among them, CD163, a scavenger receptor for the hemoglobin-haptoglobin complex, probably functions as the primary receptor for PRRSV. CD163 is mainly expressed in macrophages (Kristiansen *et al.* 2001) and is used as a phenotypic marker of M2 macrophages (Hu *et al.* 2017). In addition, CD169, which is also known as sialoadhesin or sialic acid-binding immunoglobulin-like lectin-1 (Siglec-1), is exclusively expressed on specific subpopulations of macrophages (Delputte *et al.* 2011) and is considered to be an accessory protein involved in the infection process of the PRRSV.

The expression of CD163 seems to be regulated by a number of factors in vitro (Etzerodt and Moestrup 2013). In particular, dexamethasone (DEX), a steroidal anti-inflammatory drug, is known to be a potent stimulator of the expression of CD163 in human and rodent macrophages (Schaer *et al.* 2002). Although the activity of DEX is mediated by the endogenous glucocorticoid receptor, the intracellular signaling pathways through which DEX regulates CD163 expression in macrophages are not fully understood.

In this study, to further elucidate the characteristics of IPKM, we investigated the effects of DEX treatment on CD163 and CD169 expression in IPKM. Our findings demonstrate that DEX markedly enhances CD163 expression and differentially regulates the expression of CD163 and CD169 in IPKM.

The IPKM were routinely cultured, as described previously (Takenouchi *et al.* 2017). The cells were seeded in 8-well chamber slides (2 × 10^5^ cells/well) and treated with water-soluble DEX (FUJIFILM Wako Pure Chemical Corporation, Osaka, Japan) in growth medium at the indicated concentrations. After being cultured for 1 or 3 days, they were fixed using 4% paraformaldehyde phosphate buffer solution (Nacalai Tesque, Inc., Kyoto, Japan) and immunostained using mouse monoclonal anti-CD163 (Bio-Rad, Hercules, CA) or anti-CD169 (Bio-Rad) antibodies and the EnVision system (DAKO, Hamburg, Germany), as described previously (Takenouchi *et al.* 2017). Slides were counterstained with hematoxylin, before being coverslipped. The stained slides were examined under a microscope (Leica, Bensheim, Germany).

The expression levels of CD163 and CD169 were quantitatively evaluated using a flow cytometer. IPKM (1 × 10^6^) were cultured in 90-mm non-tissue culture grade plastic dishes (Sumitomo Bakelite Co., Ltd., Tokyo, Japan) for 3 d, before being treated with the indicated reagents for 1 d. Then, the cells were detached using EDTA 0.02% solution (Sigma, St Louis, MO) and re-suspended in Dulbecco’s phosphate-buffered saline (DPBS) (1 × 10^5^ cells/100 μl) containing mouse monoclonal anti-CD203a (Bio-Rad), anti-CD163, or anti-CD169 antibodies. The number of harvested cells was measured using Bio-Rad TC10 automated cell counter. The cells were further labeled with Alexa Fluor 488-conjugated anti-mouse IgG antibodies (Invitrogen, Carlsbad, CA), before the number of Alexa Fluor 488-labeled cells was analyzed using the BD Accuri™ C6 Plus flow cytometer (BD Biosciences, Franklin Lakes, NJ). The fluorescence of 40,000 cells was assessed in each experiment. The positive region was set up to contain about 0.5% of the negative control cells, which were reacted with Alexa Fluor 488-conjugated anti-mouse IgG antibodies alone. The data on the percentages of cells in the positive region are expressed as mean±SEM values (*n* = 3). Mean values were analyzed with one-way ANOVA followed by Dunnett’s post hoc test using the software GraphPad InStat 3 for Windows. Statistical significance was set at *p* < 0.05.

IPKM were also seeded in a 24-well plate (3 × 10^5^ cells/well) and pretreated with the indicated reagents for 1 d. Then, the cells were exposed to the Fostera PRRS vaccine strain (Zoetis, Parsippany, NJ) for 2 h, washed with DPBS, and incubated for a further day. The cells were lysed with 200 μl mammalian protein extraction buffer (GE Healthcare, Chicago, IL). The level of the nucleocapsid (N) protein, a component of PRRSV particles, was evaluated by performing immunoblotting using rabbit polyclonal anti-PRRSV N protein antibodies (GeneTex, Inc.), as described previously (Takenouchi *et al.* 2011). Actin was used as an internal control for the immunoblotting. The target protein was revealed using the ECL Select reagent (GE Healthcare) and detected using a C-DiGit blot scanner (LI-COR, Inc.).

## Results and Discussion

**Figure 1.**
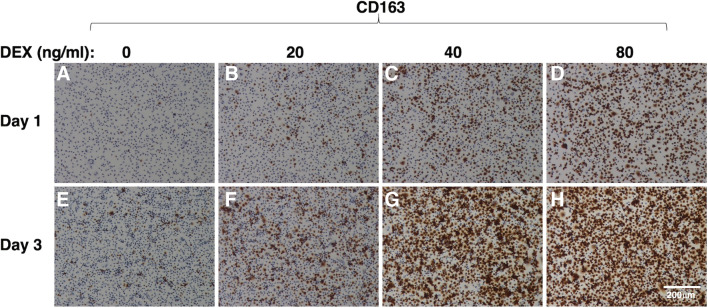
Effect of DEX on CD163 expression in IPKM. IPKM were treated with DEX at the indicated concentrations for 1 d (*A*–*D*) or 3 d (*E*–*H*). Then, the cells were fixed and immunostained with a specific antibody against CD163 (*brown*). All nuclei were counterstained with hematoxylin (*blue*). DEX increased the expression of CD163 in a dose-dependent manner.

**Figure 2.**
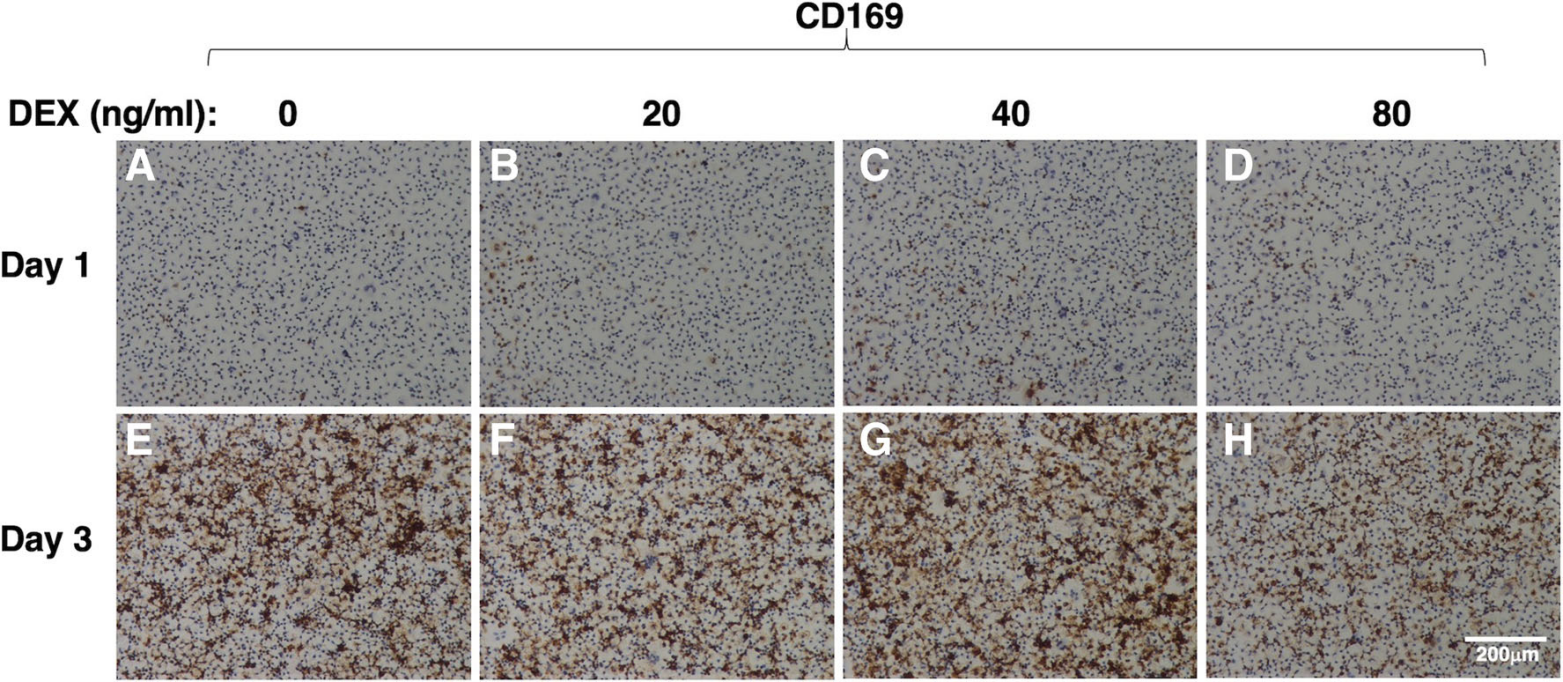
Effect of DEX on CD169 expression in IPKM. IPKM were treated with DEX at the indicated concentrations for 1 d (*A*–*D*) or 3 d (*E*–*H*). Then, the cells were fixed and immunostained with a specific antibody against CD169 (*brown*). All nuclei were counterstained with hematoxylin (*blue*). DEX did not increase the expression of CD169.

Immunostaining revealed that a few cells were significantly positive for CD163 at 1 d after plating (Fig. 1*A*). Treatment with DEX increased the number of CD163-positive cells in a dose-dependent manner (Fig. 1*A*–*D*). The number of CD163-positive cells was increased after 3 d of culturing due to cell proliferation (Fig. 1*E*). Marked DEX-induced enhancement of CD163 expression was observed at 3 d after plating (Fig. 1*E*–*H*). As was the case with CD163 expression, a few IPKM expressed CD169 at 1 d after plating (Fig. 2*A*), and the number of CD169-positive cells was increased after 3 d of culturing (Fig. 2*E*). However, in contrast to CD163 expression, treatment with DEX did not enhance the expression of CD169 (Fig. 2*A*–*D*). In fact, CD169 expression was slightly decreased after treatment with 80 ng/ml DEX (Fig. 2*H*). These findings suggest that the expression levels of CD163 and CD169 are differentially regulated by the activation of endogenous glucocorticoid receptors in IPKM.

**Figure 3.**
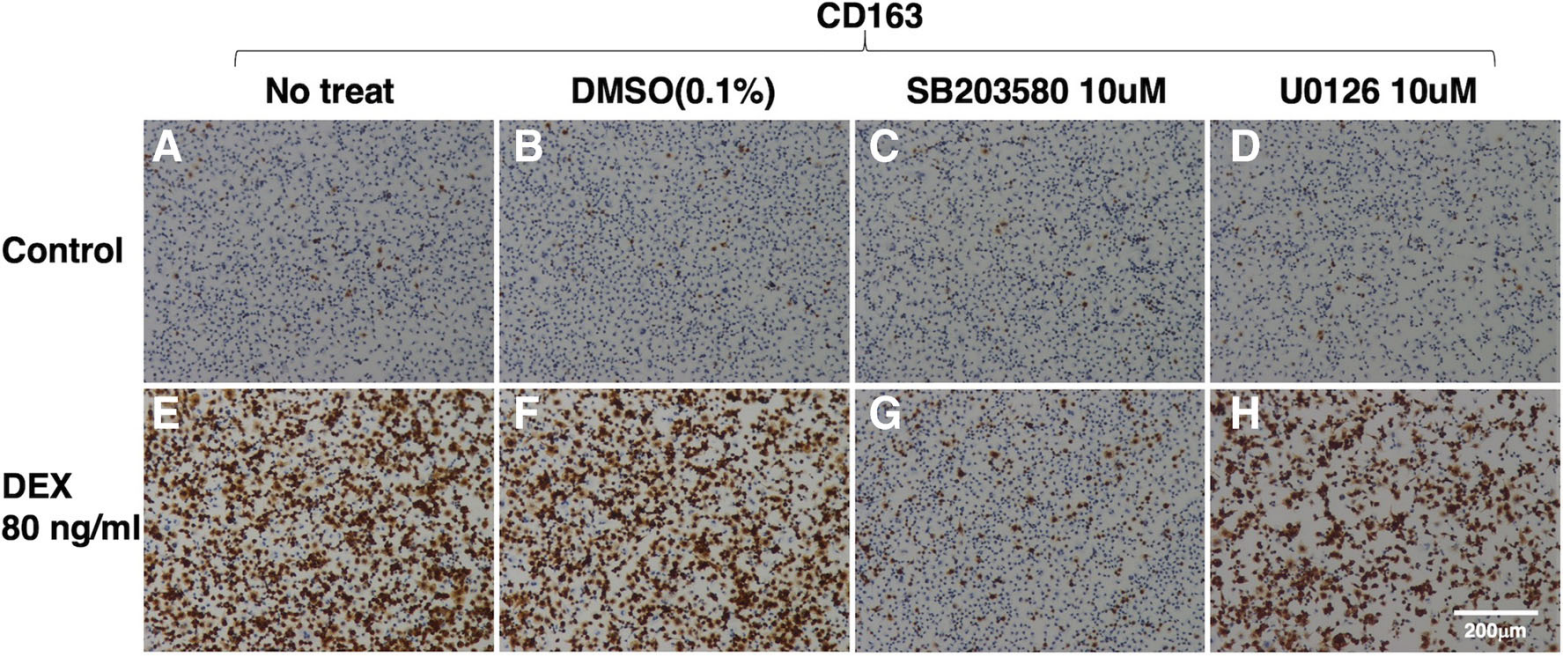
Effects of SB203580 and U0126 on the DEX-induced upregulation of CD163 expression. IPKM were treated with (*A*–*D*) or without (*E*–*H*) 80 ng/ml DEX for 1 d in the presence or absence of 10 μM SB203580 (*C*, *G*) or 10 μM U0126 (*D*, *H*). DMSO (0.1%) was used as a vehicle control (*B*, *F*) because SB203580 and U0126 were dissolved in DMSO. Then, the cells were fixed and immunostained with a specific antibody against CD163 (*brown*). All nuclei were counterstained with hematoxylin (*blue*). DEX enhanced the expression of CD163 compared with that seen in the untreated cells (*A*, *E*), even in the presence of DMSO (*B*, *F*). SB203580 and U0126 had no effect on the basal CD163 expression level (*C*, *D*). However, SB203580 blocked the DEX-induced enhancement of CD163 expression (*G*), while U0126 did not exert any such inhibitory effect (*H*).

**Figure 4.**
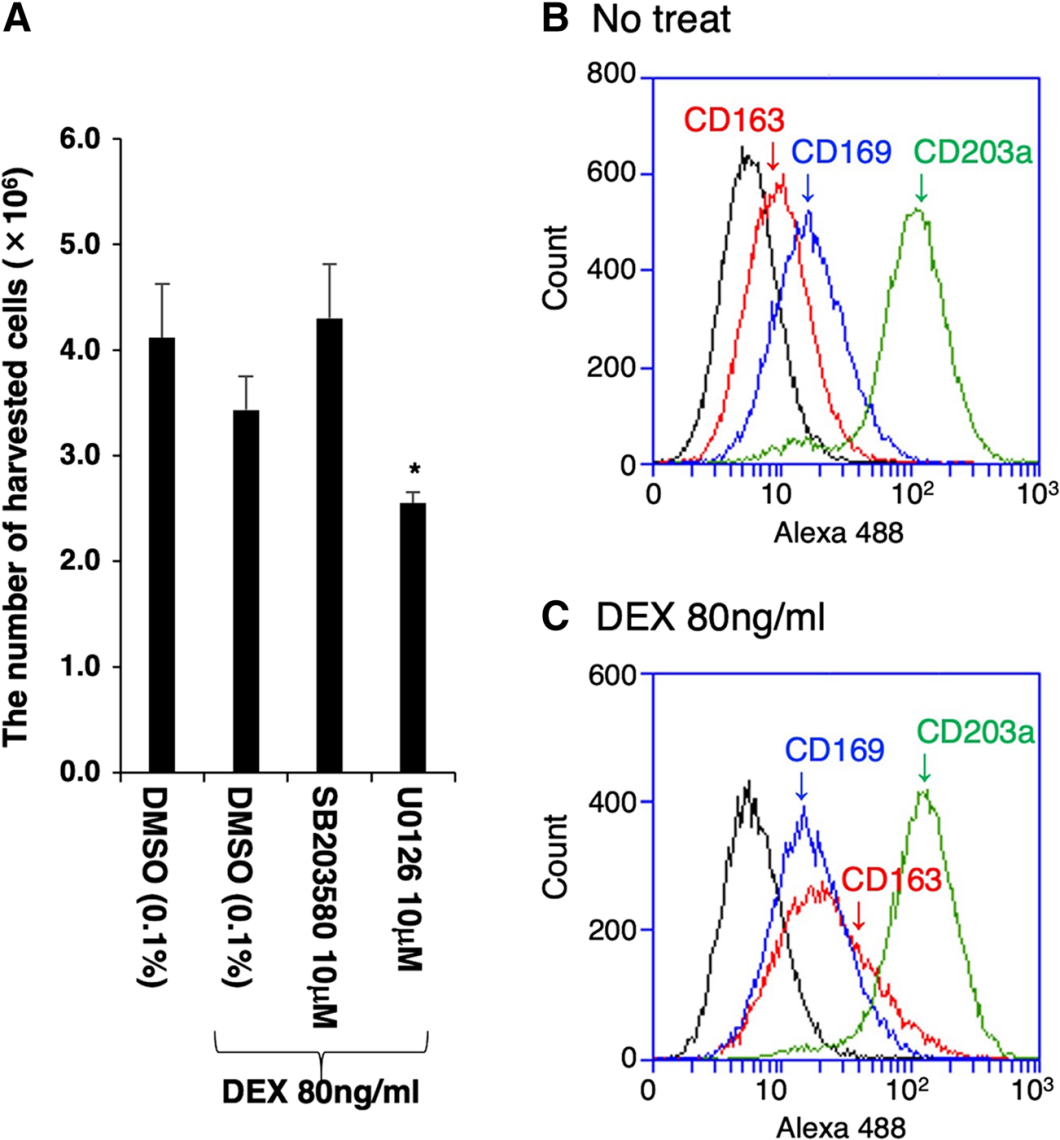
Effects of SB203580 and U0126 on the proliferation of DEX-treated IPKM and flow cytometric analyses of IPKM. IPKM (1 × 10^6^) were pre-cultured in 90-mm dishes for 3 d, and treated with or without 80 ng/ml DEX for 1 d in the presence or absence of 10 μM SB203580 or 10 μM U0126. DMSO (0.1%) was used as a vehicle control because SB203580 and U0126 were dissolved in DMSO. Then, the cells were harvested and counted with a TC10 automated cell counter (*A*). Six independent experiments were performed, and the cell numbers are expressed as mean±SEM values (*p < 0.05 vs. DMSO-treated control) (*A*). Also, no treated (*B*) and DEX-treated (*C*) IPKM were reacted with mouse monoclonal anti-CD163 (*red line*), anti-CD169 (*blue line*), or anti-CD203a (*green line*) antibodies, before being labeled with Alexa Fluor 488-conjugated anti-mouse IgG antibodies (Alexa 488). The negative control cells were reacted with Alexa Fluor 488-conjugated anti-mouse IgG antibodies alone (*B* and *C*: *black line*). The frequency of CD163-positive cells was higher (*B* and *C*, *red line*), while the frequency of CD169-positive cells was slightly lower (*B* and *C*, *blue line*), among the DEX-treated IPKM. DEX had no effect on the frequency of CD203a-positive cells (*B* and *C*, *green line*).

**Figure 5.**
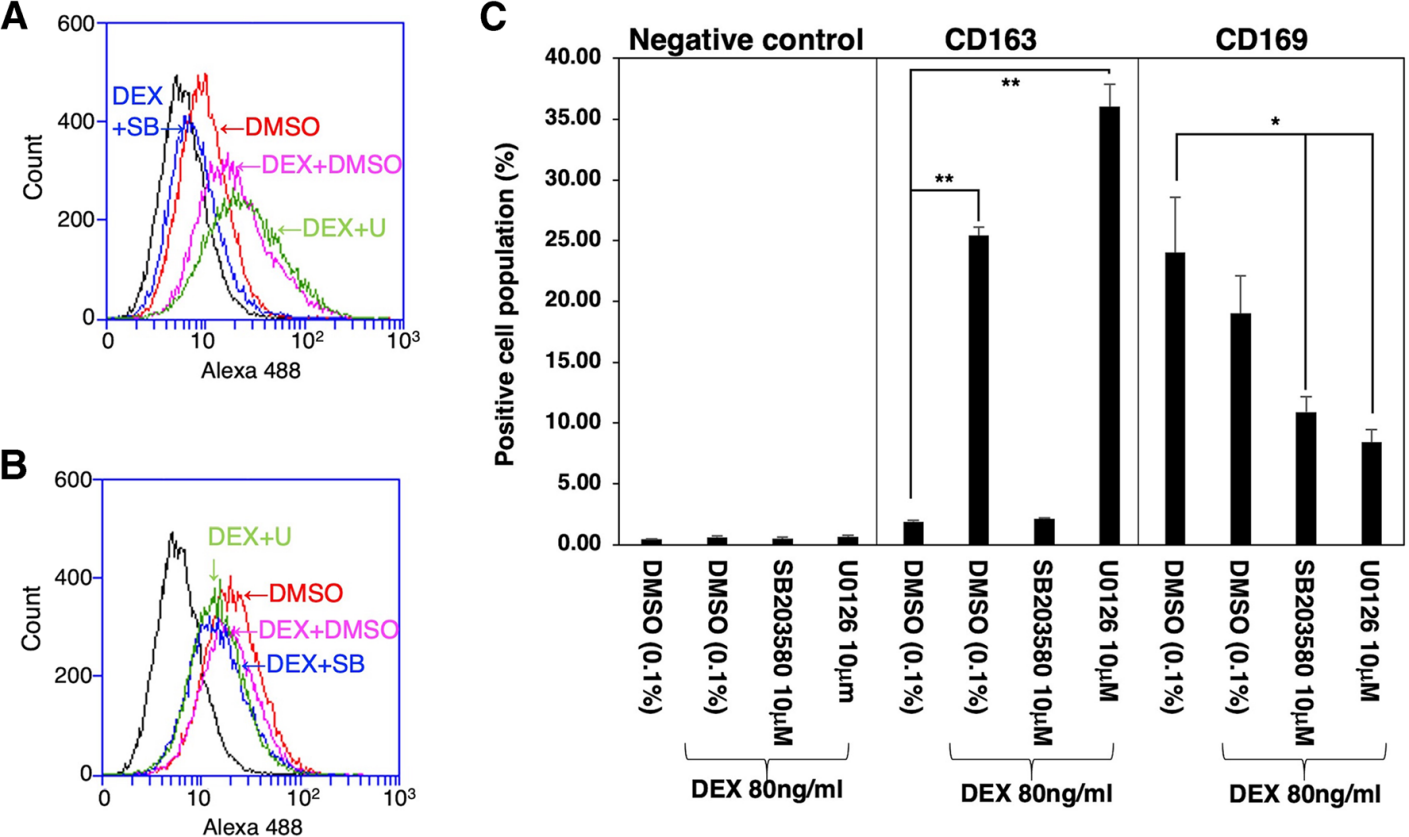
Flow cytometric analyses of DEX-treated IPKM. IPKM were treated with or without 80 ng/ml DEX for 1 d in the presence or absence of 10 μM SB203580 or 10 μM U0126. DMSO (0.1%) was used as a vehicle control because SB203580 and U0126 were dissolved in DMSO. Then, the cells were reacted with mouse monoclonal anti-CD163 (*A*) or anti-CD169 (*B*) antibodies, before being labeled with Alexa Fluor 488-conjugated anti-mouse IgG antibodies (Alexa 488). The positive region was set up to contain about 0.5% of the negative control cells, which reacted with Alexa Fluor 488-conjugated anti-mouse IgG antibodies alone (*A* and *B*: *black line*). Three independent experiments were performed, and the data regarding the percentages of cells in the positive region are expressed as mean±SEM values (*p < 0.05, **p < 0.01 vs. DMSO-treated control) (*C*). The frequency of CD163-positive cells was significantly higher among the DEX-treated IPKM (*A*: *purple line*, and *C*). The administration of SB203580 completely blocked the effects of DEX (*A*: *blue line*, and *C*). Conversely, the administration of U0126 facilitated the DEX-induced increase in the frequency of CD163-positive cells (*A*: *green line*, and *C*). The frequency of CD169-positive cells was slightly lower among the DEX-treated IPKM (*B*: *purple line*, and *C*). Co-treatment with DEX and SB203580 (*B*: *blue line*) or U0126 (*B*: *green line*) significantly decreased the frequency of CD169-positive cells (*C*).

To determine the intracellular signaling pathways through which DEX enhances CD163 expression, the effects of two kinase inhibitors, SB203580, a selective inhibitor of p38 MAPK, and U0126, a selective inhibitor of MAPK extracellular signaling-regulated kinase (ERK) kinase, were examined. These inhibitors had no effect on the basal level of CD163 expression (Fig. 3*A*–*D*), although U0126 significantly inhibited the proliferation of DEX-treated cells (Fig. 4*A*). However, SB203580 blocked the DEX-induced enhancement of CD163 expression (Fig. 3*G*), while U0126 did not exert any such inhibitory effect (Fig. 3*H*). Furthermore, the effects of SB203580 and U0126 were quantitatively assessed by flow cytometry. As a marker of mature tissue macrophages, IPKM exclusively expressed CD203a, and DEX had no effect on the frequency of CD203a-positive cells (Fig. 4*B* and *C*, green line). The frequency of CD163-positive cells was markedly higher among the DEX-treated IPKM (Fig. 4*B* and *C*, red line; Fig. 5*A*, purple line, and Fig. 5*C*). The administration of SB203580 completely blocked this effect of DEX (Fig. 5*A*, blue line, and Fig. 5*C*). Conversely, the administration of U0126 facilitated the DEX-induced increase in the frequency of CD163-positive cells (Fig. 5*A*, green line, and Fig. 5*C*). On the other hand, the frequency of CD169-positive cells was slightly lower among the DEX-treated IPKM (Fig. 4*B* and *C*, blue line; Fig. 5*B*, purple line, and Fig. 5*C*). Co-treatment with DEX and SB203580 (Fig. 5*B*, blue line) or U0126 (Fig. 5*B*, green line) significantly decreased the frequency of CD169-positive cells (Fig. 5*B* and *C*). These findings agreed with the immunostaining data.

**Figure 6.**
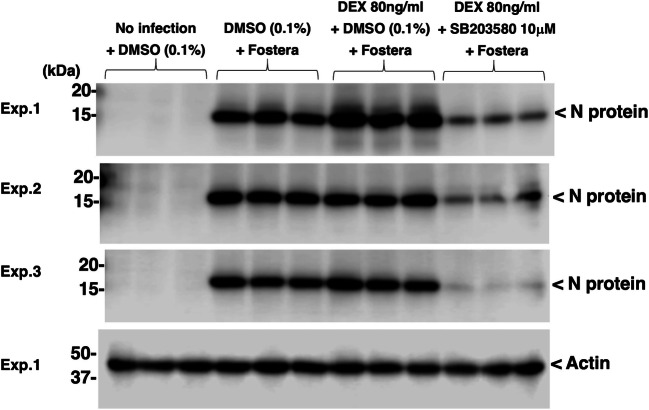
Detection of N protein in Fostera PRRSV–infected IPKM. IPKM were treated with or without 80 ng/ml DEX for 1 d in the presence or absence of 10 μM SB203580. DMSO (0.1%) was used as a vehicle control because SB203580 was dissolved in DMSO. Then, the cells were exposed to the Fostera PRRSV for 2 h, cultured for a further day, and lysed with extraction buffer. The N protein was detected in the Fostera PRRSV-exposed IPKM, even in the presence of DMSO. DEX treatment had no effect on the expression of the N protein, while SB203580 clearly reduced it. The experiments were performed in triplicate and produced similar results (*upper three panels*). An anti-actin antibody was used as a loading control for the immunoblotting (*the lowest panel*).

As CD163 is considered to be a putative receptor for the PRRSV (Calvert *et al.* 2007), we investigated the effects of DEX on PRRSV infection in IPKM. To this end, Fostera PRRS, an attenuated PRRSV vaccine strain (Park *et al.* 2015), was used because this PRRSV strain can be passaged in baby hamster kidney (BHK) cells that have been engineered to express porcine CD163. Immunoblotting clearly detected the PRRSV N protein in the IPKM lysate on day 1 after infection (Fig. 6), indicating that the Fostera PRRSV had been successfully propagated in these cells. Contrary to our expectations, pretreatment with DEX did not affect Fostera PRRSV propagation (Fig. 6), suggesting that DEX-enhanced CD163 expression might not play a critical role in the promotion of PRRSV propagation. Interestingly, pretreatment with SB203580 prevented the Fostera PRRSV infection of DEX- treated IPKM (Fig. 6).

It was reported that DEX stimulates p38 MAPK phosphorylation, which leads to lymphoid cell apoptosis (Miller *et al.* 2005). DEX is also reported to induce the phosphorylation of p38 MAPK in osteoblast-like MC3T3-E1 cells, which is inhibited by SB203580 (Kozawa *et al.* 2002). We previously demonstrated that SB203580 blocked the DEX-dependent induction of adipogenesis in 3T3-L1 cells (Takenouchi *et al.* 2004). These findings support the notion that DEX might stimulate p38 MAPK phosphorylation in IPKM, resulting in the induction of CD163 transcription. However, the DEX treatment conditions required to stimulate p38 MAPK phosphorylation in IPKM have not yet been determined.

Since the induction of CD163 expression confers susceptibility to the PRRSV in several non-permissive cell lines (Calvert *et al.* 2007), we hypothesized that DEX-induced enhancement of CD163 expression increases the efficiency of PRRSV infection. However, DEX treatment did not affect the propagation of the Fostera PRRSV in IPKM. This suggests that DEX-induced enhancement of CD163 expression alone is not sufficient to facilitate the infection of IPKM by PRRSV. In addition, we found that CD169 expression tends to be suppressed by DEX. It is speculated that the expression patterns of not only CD163 and CD169 but also those of other PRRSV receptors affect the process by which PRRSV infects IPKM. Actually, a previous study demonstrated that DEX treatment enhanced the expression of both CD163 and CD169 in porcine monocytes, which led to an increase in the susceptibility of these cells to infection by PRRSV-1 strain Luna (Singleton *et al.* 2018). Therefore, a DEX-induced reduction in CD169 expression in IPKM might result in DEX having no effect on the susceptibility of these cells to infection by the Fostera PRRSV.

## Conclusion

In summary, we demonstrated that DEX treatment enhanced the expression of CD163 in IPKM, which might be regulated by p38 MAPK-dependent pathways. Although DEX treatment had no effect on the propagation of the PRRSV in IPKM, the administration of SB203580 prevented the propagation of the virus, suggesting that the p38 MAPK signaling pathway might be involved in the process responsible for PRRSV propagation. Further studies are required to examine this intriguing possibility. The present study provides additional information about the unique features of IPKM and supports their usefulness as a model of porcine macrophages.
